# Cascaded spintronic logic with low-dimensional carbon

**DOI:** 10.1038/ncomms15635

**Published:** 2017-06-05

**Authors:** Joseph S. Friedman, Anuj Girdhar, Ryan M. Gelfand, Gokhan Memik, Hooman Mohseni, Allen Taflove, Bruce W. Wessels, Jean-Pierre Leburton, Alan V Sahakian

**Affiliations:** 1 Department of Electrical Engineering & Computer Science, Northwestern University, 2145 Sheridan Road, Evanston, Illinois 60208, USA; 2 Department of Electrical & Computer Engineering, The University of Texas at Dallas, 800 W. Campbell Road, Richardson, Texas 75080, USA; 3 Department of Physics, University of Illinois at Urbana-Champaign, 405 North Mathews Avenue, Urbana, Illinois 61801, USA; 4 Beckman Institute for Advanced Science & Technology, University of Illinois at Urbana-Champaign, 405 North Mathews Avenue, Urbana, Illinois 61801, USA; 5 CREOL, The College of Optics and Photonics, University of Central Florida, 4304 Scorpius Street, Orlando, Florida 32816, USA; 6 Department of Materials Science & Engineering, Northwestern University, 2145 Sheridan Road, Evanston, Illinois 60208, USA; 7 Department of Electrical & Computer Engineering, University of Illinois at Urbana-Champaign, 405 North Mathews Avenue, Urbana, Illinois 61801, USA; 8 Department of Biomedical Engineering, Northwestern University, 2145 Sheridan Road, Evanston, Illinois 60208, USA

## Abstract

Remarkable breakthroughs have established the functionality of graphene and carbon nanotube transistors as replacements to silicon in conventional computing structures, and numerous spintronic logic gates have been presented. However, an efficient cascaded logic structure that exploits electron spin has not yet been demonstrated. In this work, we introduce and analyse a cascaded spintronic computing system composed solely of low-dimensional carbon materials. We propose a spintronic switch based on the recent discovery of negative magnetoresistance in graphene nanoribbons, and demonstrate its feasibility through tight-binding calculations of the band structure. Covalently connected carbon nanotubes create magnetic fields through graphene nanoribbons, cascading logic gates through incoherent spintronic switching. The exceptional material properties of carbon materials permit Terahertz operation and two orders of magnitude decrease in power-delay product compared to cutting-edge microprocessors. We hope to inspire the fabrication of these cascaded logic circuits to stimulate a transformative generation of energy-efficient computing.

## Abstract (web summary)

Spintronics, graphene, and carbon nanotubes are potential components of next-generation high performance computers. Here, the authors propose and theoretically evaluate a spintronic logic family composed solely of carbon materials with the potential for a 100 × improvement in energy efficiency.

Manipulation of the spin-degree of freedom for spintronic computing requires the invention of unconventional logic families to harness the unique mechanisms of spintronic switching devices^1^,^2^,^3^,^4^,^5^,^6^,^7^,^8^,^9^,^10^,^11^,^12^,^13^,^14^. Cascading, one device directly driving another device, has been well known as a major challenge and fundamental requirement of a logic family since von Neumann's^15^ 1945 proposal for a stored-program electronic computer. If the input and output signals are not of the same type and magnitude, it is difficult to connect devices without an additional device for translation. This extra device consumes power, time and area, and severely degrades the utility of the logic family.

Here we present an alternative paradigm for computing: all-carbon spin logic. This cascaded logic family creatively applies recent nanotechnological advances to efficiently achieve high-performance computing using only low-dimensional carbon materials^16^,^17^,^18^,^19^,^20^,^21^,^22^,^23^,^24^. A spintronic switching device is proposed utilizing the negative magnetoresistance of graphene nanoribbon (GNR) transistors^25^,^26^,^27^,^28^,^29^ and partially unzipped carbon nanotubes (CNTs)^30^,^31^, unzipped^30^,^32^,^33^,^34^,^35^ from metallic CNT interconnect. These carbon gates can be cascaded directly; no additional intermediate devices are required between logic gates. The physical parameters necessary for proper switching operation are evaluated through mean-field tight-binding calculations of the band structure to enable an analysis of computational efficiency and to provide guidance for an experimental proof of concept. The results demonstrate the potential for compact all-carbon spin logic circuits with Terahertz operating speeds and two orders of magnitude improvement in power-delay product, thus motivating further investigation of the proposed device and computational structure.

## Results

### Device structure and physical operation

**Figure 1:**
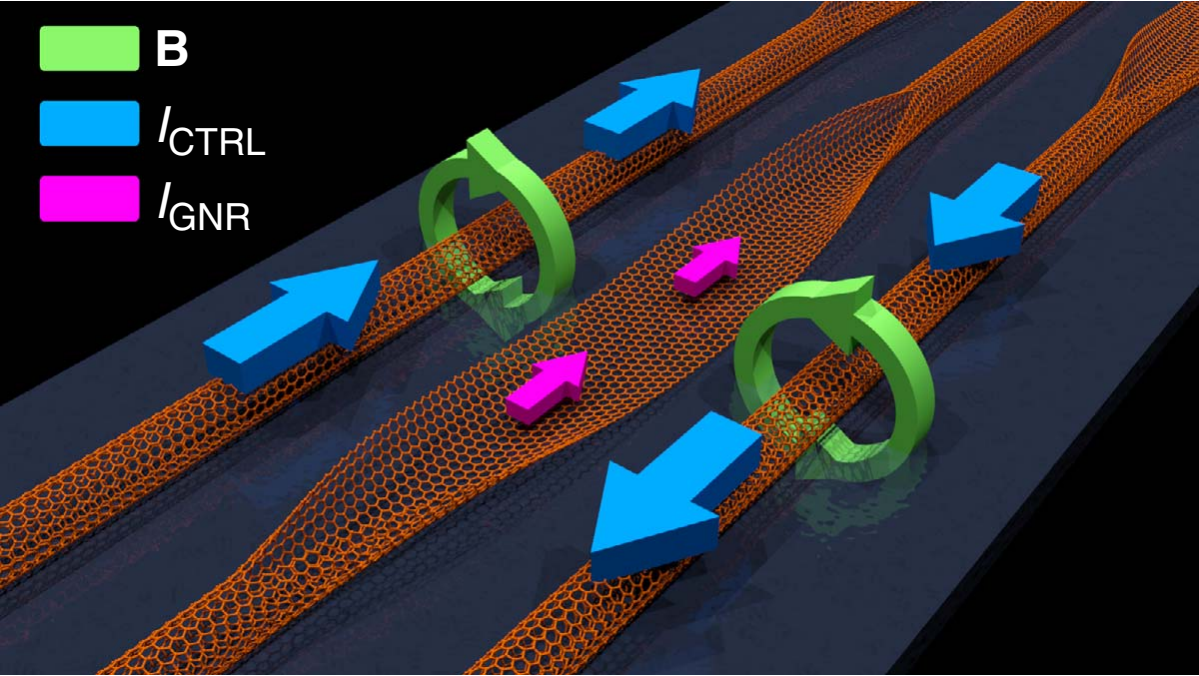
All-carbon spin logic gate. Magnetoresistive GNR unzipped from carbon nanotube and controlled by two parallel CNTs on an insulating material above a metallic gate. As all voltages are held constant, all currents are unidirectional. The magnitudes and relative directions of the input CNT control currents *I*_CTRL_ determine the magnetic fields **B** and GNR edge magnetization, and thus the magnitude of the output current *I*_GNR_.

**Figure 2:**
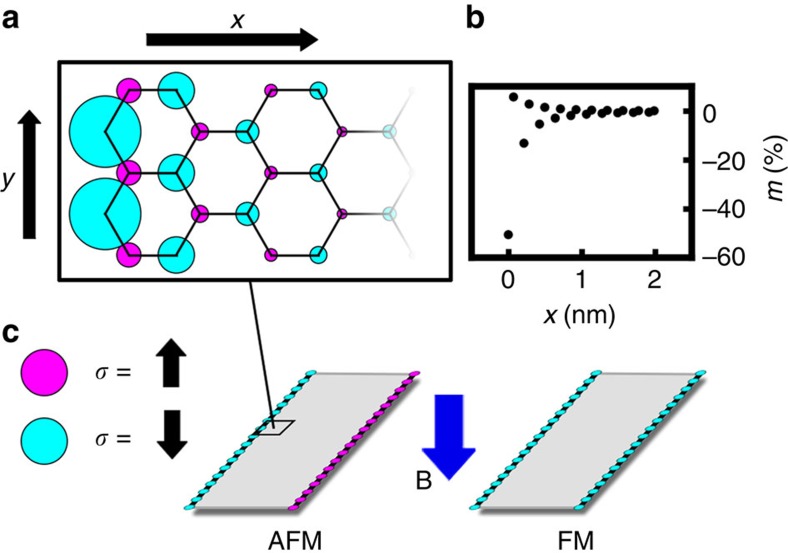
Graphene nanoribbon edge magnetization. (**a**) On-site magnetization profile of a zigzag graphene edge. The magnetic field created by an adjacent CNT current causes strong on-site magnetization at the GNR edge. The colour of each circle represents the spin species, while the radius corresponds to the magnitude of the magnetization. (**b**) The on-site magnetization of each site in a unit cell as a function of distance from the edge. (**c**) Graphene nanoribbon edge magnetization in the absence and presence of an externally applied magnetic field. In the absence of a magnetic field, the GNR exhibits global AFM ordering with edges of opposite polarities. The application of a magnetic field aligns the edge polarities, achieving global FM ordering.

The active switching element is a zigzag GNR field-effect transistor with a constant gate voltage and two CNT control wires, as illustrated in Fig. 1. The gate voltage is held constant, and the GNR conductivity is therefore modulated solely by the magnetic fields generated by the CNTs. These magnetic fields can flip the orientation of the strong on-site magnetization at the GNR edges, which display local antiferromagnetic (AFM) ordering due to Hubbard interactions^36^ (see Methods). As shown in Fig. 2, the magnetization at each edge is controlled by its neighbouring CNT, with magnetization decaying towards the centre of the GNR. In the absence of an external magnetic field or with edge magnetizations of opposite polarities, the GNR exhibits global AFM ordering in the ground state. Significantly, GNRs with edge magnetizations of the same polarity exhibit global ferromagnetic (FM) ordering in the ground state.

Mean-field tight-binding calculations show that the GNR global magnetic ordering determines the band structure, and therefore the conductivity. The Zeeman interaction can switch the magnetic ground state, causing spin-dependent band splitting. There are conduction modes in the FM state for all energies, but no conduction modes in the AFM state for Fermi energy *E*_F_ within the AFM state bandgap. By tuning *E*_F_ into the AFM bandgap through control of the gate voltage, the application of magnetic fields at the GNR edges causes a colossal change in conductivity, switching the GNR from the resistive AFM state to a conductive FM state. Importantly, if *E*_F_ is outside the AFM bandgap, conduction modes are always available, and switching of the magnetic ordering does not cause a change in conductivity. This is one possible explanation for the lack of magnetoresistance observed by Bai *et al*.^28^ when applying an in-plane magnetic field to a GNR. It can be further noted that given the proximity between the CNTs and GNR, the attractive van der Waals and repulsive Casimir forces may alter the electronic wavefunctions and energy dispersion. However, these effects do not change the nature of the highly conductive CNT transport, nor the electron–electron repulsion among lattice sites. As a result, the spontaneous AFM ordering and edge magnetization are not sufficiently affected to alter the GNR magnetoresistance.

### Edge effects and operation temperature

**Figure 3:**
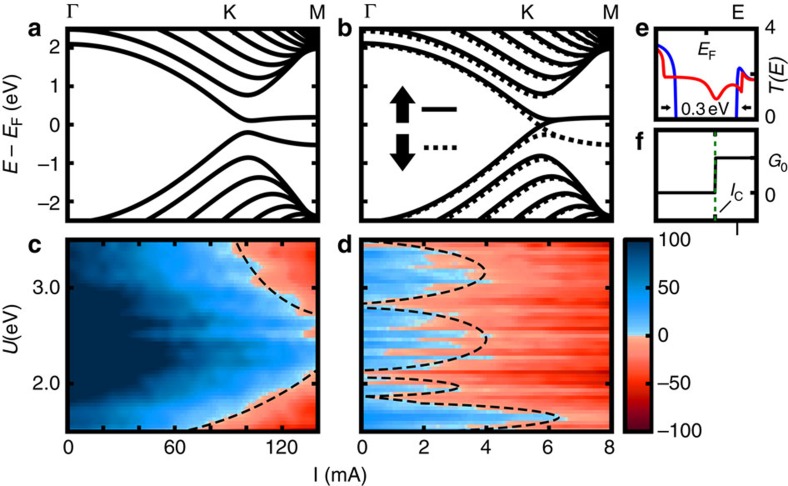
Magnetoresistive behaviour of GNR controlled by adjacent CNTs. (**a**,**b**) Band diagrams for the AFM and FM global ordering of a 12-atom-wide zigzag GNR with zero current in the CNTs and Hubbard parameter *U*=2.7 eV, as in equation (1) of Methods. In the global AFM state (**a**), there is a large gap between the valence and conduction bands, within which lies the Fermi energy, *E*_F_. Therefore, there are no available conduction modes, and the conductance is zero. In the FM state (**b**), there is no bandgap and there is at least one conduction mode at all energies. (**c**,**d**) The magnetic instability energy in μeV for zigzag GNRs with widths of (**c**) 20 nm and (**d**) 35 nm. The blue region designates a positive instability energy (the insulating AFM state), while the red region indicates negative instability energies (the conductive FM state). In the narrower GNR transistor, the axes of the CNTs are 10 nm from the GNR edge, while the wider GNR has CNTs placed 1 nm away. The critical switching current, which depends on *U*, is denoted with a dashed line. (**e**) The transmission function 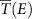 of the AFM state defines the number of available conduction modes as well as the probability for an electron to travel across the device. Thus, for *E*_F_ values within the bandgap, the GNR conductance switches when the global ordering switches between the FM and AFM states. (**f**) A typical switching event, where the GNR conductance increases by *G*_0_ when the CNT current overcomes the critical switching current *I*_C_.

The spin-dependent band splitting is strongest with pristine zigzag edges^37^ as achieved in ref. 35, enabling a spin-polarized current^38^ and closing the energy gap for the GNR in the FM state as shown in Fig. 3. Local edge defects in quasi-pristine GNRs cause local perturbation in the magnetic ordering around these defects^37^. However, the magnetic state is quickly regained within two unit cells (<1 nm) from the defect. As a result, the switching mechanism persists with defect spacing >3 nm, increasing the magnitude of the critical magnetic field for large defect density. As the magnetic ordering originates from the GNR edges, it is not affected significantly by defects present away from the edges. In the case of very rough edges, a lack of sufficient contiguous zigzag portions to compensate for the presence of armchair edges may result in large switching fields.

Smooth GNRs with long contiguous stretches of pristine zigzag edges have been experimentally demonstrated^35^. As defects affect the magnetization on the order of 1 nm around the defect location^37^, this abundance of zigzag edges of 5 nm or larger elicits strong magnetic ordering. Sufficient contiguous zigzag edges between defects thus enable a persistence of the magnetic order.

Yazyev^39^ indicated a GNR Curie temperature near 10 K, below which the spin correlation length grows exponentially. At temperatures around 70 K, correlation lengths are on the order of 10 nm, presenting a limitation for device operation. The correlation length approaches 1 nm at room temperature, making observation of the magnetization difficult in disordered systems. Therefore, low temperatures are desirable to minimize the required magnetic field and to ensure the manifestation of this effect in large samples. This concern may have been resolved, with magnetic order recently demonstrated in zigzag GNRs at room temperature^40^.

### Switching behaviour

We performed simulations of the proposed all-carbon spintronic switching device to determine the system and material parameters required to ensure feasibility. The magnetic instability energy is dependent on the GNR width (Supplementary Note 1), and determines the edge magnetic field required to switch the global ground state from AFM to FM ordering. As shown in Fig. 3c,d, the CNT current sufficient to overcome the magnetic instability energy is strongly affected by the proximity of the CNT control wires to the GNR edges. The current requirement can be tuned through control of the Hubbard *U* parameter. The required current ranges from exceptionally small magnitudes to significant fractions of an Ampere, and can be minimized with a wide GNR positioned close to the CNT control wires. As the GNR width is increased, the magnetic instability energy decreases as nearly the inverse square of the width^25^. For many *U* values and GNR/CNT geometries, the 20 μA that can be passed through a single-walled CNT is sufficient to maintain the required switching current^41^,^42^,^43^.

When the GNR switches from the AFM to the FM state, there is a massive change in conductance, as shown in Fig. 3f. The magnitude of the current through the GNR functions as the binary gate output, with binary 1 representing the large current of the conductive FM state and binary 0 representing the resistive AFM state. The GNR current flows through the CNT from which it was unzipped, and this binary CNT current is the input to cascaded GNR gates. It should be noted that unlike other spintronic logic proposals, logic gates can be cascaded directly through the carbon materials without requiring intermediate control or amplification circuitry.

### Logic gates and system integration

The various combinations of input magnitudes and directions permit the computation of the logical OR and XOR operations. When there is no difference in magnetization between the edges of the GNR, the GNR is in the resistive AFM state and outputs a binary 0. Application of current through the CNTs can cause the GNR to switch into the FM state and output a binary 1. The OR logic function of Table 1 is computed by CNT currents oriented in opposite directions that create aligned on-site magnetization at the GNR edges. This OR gate thus enables a highly conductive FM state in the presence of current in at least one input CNT. In the XOR logic function of Table 2, the input currents are oriented in the same direction. Therefore, large currents flowing through both CNTs cause AFM ordering in the XOR gate, resulting in a small output current. This GNR switching device provides the functionality necessary for general-purpose computing, as the OR and XOR gates form a sufficient basis set to generate all binary functions.

**Figure 4:**
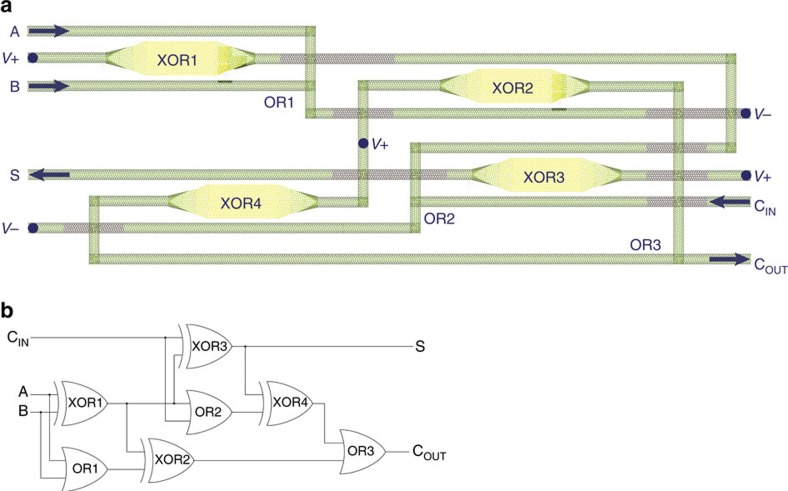
All-carbon spin logic one-bit full adder. (**a**) The physical structure of a spintronic one-bit full adder with magnetoresistive GNR FETs (yellow) partially unzipped from CNTs (green), some of which are insulated (brown) to prevent electrical connection. The all-carbon circuit is placed on an insulator above a metallic gate with constant voltage *V*_G_. Binary CNT input currents A and B control the state of the unzipped GNR labelled XOR1, which outputs a current with binary magnitude 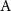
⊕B. The output of XOR1 flows through a CNT that functions as an input to XOR2 and XOR3 before reaching the wired-OR gate OR2, which merges currents to compute C_IN_V(A⊕B). This current controls XOR4 and terminates at *V*_−_. The other currents operate similarly, computing the one-bit addition function with output current signals S and C_OUT_. (**b**) In the symbolic circuit diagram shown here with conventional symbols, the output of XOR1 is used as an input to OR2 and XOR3 along with *C*_IN_. The full adder S output is computed as S=C_IN_V⊕(A⊕B). OR2 outputs C_IN_V(A⊕B), which is used along with S as an input to XOR4 to compute (C_IN_V(A⊕B))⊕(C_IN_⊕(A⊕B)). This output of XOR4 is equivalent to (A∧C_IN_)V(B∧C_IN_). OR3 takes this signal as an input along with the output of XOR2, which is equal to A∧B, to compute C_OUT_=(A∧B)V(A∧C_IN_)V(B∧C_IN_). As the wired-OR gates simply sum the currents and have no significant delay, the total propagation time is that of three XOR gates, determined by the XOR1–XOR3–XOR4 worst-case path.

Nanofabrication trends suggest potential techniques for efficiently constructing cascaded all-carbon spin logic integrated circuits scaled up to perform complex computing tasks. Parallel and perpendicular CNTs can be laid out on an insulating surface^44^ above a metallic material used as a constant universal gate voltage for the entire circuit. As shown in Fig. 4, a complex circuit composed of the logic gates of Fig. 1 can be created through selective CNT unzipping to form GNRs^24^,^30^,^32^,^33^,^34^,^35^. Electrical connectivity between overlapping CNTs^34^,^45^,^46^ can be determined by the placement of an insulating material. The only external connections are to the supply voltage and user input/output ports (for example, keyboard, monitor and so on), possibly with vertical covalent contacts of the type described by Tour^47^. All computing functionality is performed by the carbon materials alone, without the aid of external circuitry. As in other large-scale integrated circuits, fabrication imprecision (for example, misaligned CNTs, imperfect CNT junctions, edge defects and so on) can be tolerated provided that the GNR logic gates function properly and the electrical connectivity between CNTs is correct. Though the possibility of miniaturization is an important figure of merit for conventional computing structures, the atomic dimensions of CNTs and GNRs make the concept of down-scaling irrelevant for all-carbon spin logic.

Cascaded all-carbon spin logic gates can be connected by routing the GNR output currents through the CNT control inputs of other GNR gates. Four XOR gates and three wired-OR gates are cascaded in Fig. 4 to realize a full adder, an essential computational function traditionally performed with 28 CMOS transistors (Supplementary Fig. 1 and Supplementary Table 1). The supply voltage nodes *V*_+_ and *V*_−_ are held constant, thereby causing the polarities of all current paths to be constant. Binary switching results from changes in input current magnitudes due to changes in the output currents of other gates, without amplification, conversion or control circuitry. The output currents flow through the inputs of other logic and memory elements such as parity gates and toggle latches (Supplementary Figs 2 and 3, and Supplementary Table 2). These circuits provide traditional logic functionality with far fewer devices, enabling compact spintronic computing systems.

## Discussion

While these circuits may be implemented with other materials exhibiting high conductivity and negative magnetoresistance, the exceptional properties of CNTs and GNRs make these structures ideal candidates for use in this logic family. Current is the state variable in all-carbon spin logic, enabling exceptionally fast computation with switching delay determined by electromagnetic wave propagation. This is in stark contrast to conventional computing systems in which voltage is the state variable, leading to CMOS switching and RLC interconnect delays limited by charge transfer and accumulation.

**Figure 5:**
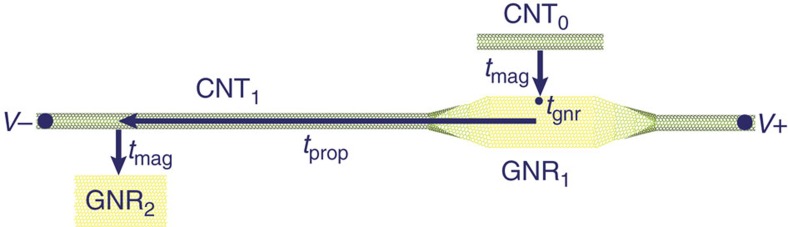
Analysis of switching and propagation delay. Following a switch in the current through CNT_0_ at a time *t*=0, the magnetic field at the edge of GNR_1_ switches at *t*=*t*_mag_, the resistance of GNR_1_ switches at *t*=*t*_mag_+*t*_gnr_ and the current through CNT_1_ switches at *t*=*t*_mag_+*t*_gnr_+*t*_prop_. This marks the end of one complete switching and propagation cycle, and is immediately followed by the switching of GNR_2_.

As described by Fig. 5, the GNR conductivity switches far faster than the signal can propagate through the CNTs^48^. The all-carbon spin logic switching time *t*_d_=*t*_mag_+*t*_gnr_+*t*_prop_ is the summation of the times required for a CNT current to switch a magnetic field in a neighbouring GNR (*t*_mag_), the GNR magnetoresistance to switch in response to a magnetic field (*t*_gnr_) and the electric field to propagate through the CNT to switch the current (*t*_prop_). The propagation time *t*_prop_ is significantly larger than *t*_mag_ and *t*_gnr_, and therefore determines *t*_d_. The electromagnetic wave propagation speed in a CNT is 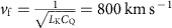, where *L*_K_=400 pH nm^−1^ is the kinetic inductance and *C*_Q_=0.4 aF nm^−1^ is the quantum capacitance^48^. For 400 nm CNT interconnect length *l*_cnt_, the worst-case logic gate switching time is 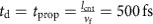. High-performance circuits operating with clock frequencies of 2 THz can therefore be realized. The average power dissipation per logic gate is 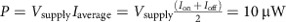, given a differential supply voltage *V*_supply_=*V*_+_−*V*_−_=1 V and an on-state current *I*_on_=*I*_C_≈20 μA sufficiently small to flow through a single-walled CNT. The power-delay product for each gate, a metric of computing efficiency, can be determined for all-carbon spin logic as PDP=*Pt*_d_≈5 × 10^−18^ J. This is approximately 100 times more energy-efficient than 22 nm CMOS.

Furthermore, the power dissipation of all-carbon spin logic is nearly independent of frequency, whereas conventional CMOS circuits dissipate increasing power as clock frequency is increased. This two orders of magnitude improvement outweighs the power costs of low-temperature operation and leaves significant room for second-order parasitic effects. This direct comparison can be made due to the absence of additional circuitry between logic gates, in contrast to other spintronic logic proposals. In addition, each GNR switching device in the all-carbon spin logic family performs the functionality of between four and twelve CMOS transistors. The one-bit full adder of Fig. 4, for example, has only four active GNR gates and a propagation time of 3*t*_d_, yielding a PDP of 6 × 10^−17^ J.

**Figure 6:**
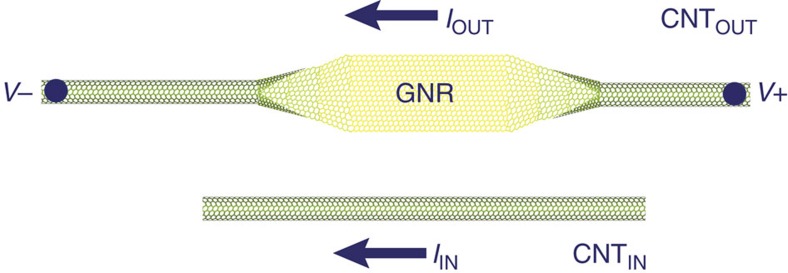
Proposed proof-of-concept experiment. By measuring the change in *I*_OUT_ in response to a change in *I*_IN_, the central component of all-carbon spin logic can be demonstrated. The carbon nanotube CNT_OUT_ can be partially unzipped such that a portion forms a GNR. A second CNT, CNT_IN_, is then placed nearly parallel to CNT_OUT_. A constant voltage should be applied across CNT_OUT_. It is not necessary to achieve the dimensions described in this work; rather, to make the experiment more facile, CNT_IN_ must merely be close enough to the GNR to cause a measurable response in *I*_OUT_ when *I*_IN_ is varied. Furthermore, as shown in the figure, CNT_IN_ need not be as long as CNT_OUT_, thereby preventing the CNTs from making contact even if the CNTs are not perfectly parallel.

All-carbon spin logic permits the development of cascaded spintronic logic circuits composed solely of low-dimensional carbon materials without intermediate circuits between gates, resulting in compact circuits with reduced area that are far more efficient than CMOS. Though a complete all-carbon spin logic system is several years away from realization, currently available technology permits experimental proof of the concept as shown in Fig. 6. By exploiting the exotic behaviour of GNRs and CNTs, all-carbon spin logic enables a spintronic paradigm for the next generation of high-performance computing.

## Methods

### Hubbard tight-binding Hamiltonian

The tight-binding Hamiltonian for a zigzag-edged GNR is


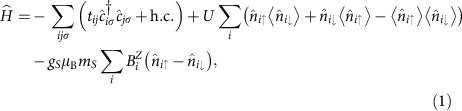


where *t*_*ij*_ is the transfer integral between orbitals localized at sites *i* and *j* of the GNR lattice, and 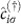 is the annihilation (creation) operator for an electron of spin *σ* at site *i*. Interactions up to the third nearest neighbour are considered, and the values for all transfer and overlap integrals are taken from set D of Hancock *et al*.^37^. In the second term, which represents the Coulomb interaction between electrons, *U* is the repulsive Hubbard parameter and 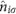
is the on-site occupation of an electron with spin *σ*. In the mean-field approximation, the expectation of the on-site occupation is used to reduce the complexity of the Hamiltonian, which can be solved with iterative methods^49^,^50^. Finally, the third term represents the Zeeman interaction, where *g*_S_ is the electron Landé g-factor, *μ*_B_ is the Bohr magneton, *m*_S_ is the z-component of electron spin and 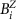 is the *z*-component of the magnetic field at site *i*. The non-homogeneous magnetic field is generated by the Biot–Savart law and permeates everywhere in space, thereby affecting every atom in the GNR (not only the edges). The magnitudes of the magnetic fields in this study are small enough to neglect phase changes in the transfer integrals due to the magnetic field.

### Diagonalization and the secular equation

By taking advantage of the translational symmetry of a GNR, the **k**^th^ component of the Hamiltonian for spin *σ* can be written as


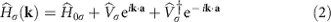


where 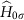 represents the interactions within one unit cell, and 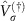 is the interaction between one cell and the next (previous) unit cell at a displacement of **a** (−**a**). Each component of the single-particle states can be calculated by solving the secular equation


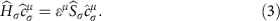


Here 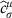 is the eigenstate of spin *σ* corresponding to the energy 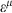 and 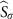 is the overlap matrix, which is the identity matrix in the investigated parameter set. The energies *ɛ*^**k**_*σ*_^ corresponding to *N* states 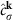 where *N* is the number of sites in the unit cell, define the band structure of the GNR. At the GNR edges, we assume hydrogen passivation *sp*^2^ dangling bonds, which is the standard treatment of edges in simulations of transport through GNRs. As there is no dangling bond, there is no reconstruction other than a slight modification of the bond angle between H–C and C–C bonds. This has a negligible effect on the magnetization of the edges. In our calculations, we use 30,000 **k** points in the Brillouin zone.

### Mean-field approximation

At zero temperature, the *N* lowest states are populated, and the occupation of the *i*^th^ site in the unit cell is given by


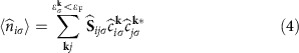


This value is used as an input to the Hamiltonian for the next iteration, and this process is repeated until the maximum change in occupation at any site is <5 × 10^−7^ per iteration.

### Data availability

Data supporting the findings of this study are available from the authors on request.

## Additional information

**How to cite this article:** Friedman, J. S. *et al*. Cascaded spintronic logic with low-dimensional carbon. *Nat. Commun.* **8**, 15635 doi: 10.1038/ncomms15635 (2017).

**Publisher's note:** Springer Nature remains neutral with regard to jurisdictional claims in published maps and institutional affiliations.

## Supplementary Material

Supplementary Information Supplementary Figures, Supplementary Tables and Supplementary Note

## Figures and Tables

**Table 1:** GNR OR gate truth table for input CNT control currents in opposite directions.

***I***_**A**_	***I***_**B**_	**State**	***I***_**O**_
0	0	AFM	0
0	1	FM	1
1	0	FM	1
1	1	FM	1

**Table 2:** GNR XOR gate truth table for input CNT control currents in the same direction.

***I***_**A**_	***I***_**B**_	**State**	***I***_**O**_
0	0	AFM	0
0	1	FM	1
1	0	FM	1
1	1	AFM	0
