# Supplementary material for: Cascaded spintronic logic with low-dimensional carbon
Source: Nat Commun. 2017 Jun 5;8:15635. doi: 10.1038/ncomms15635 (PMC5465351; doi:10.1038/ncomms15635)
Supplement: Supplementary Information — Supplementary Figures, Supplementary Tables and Supplementary Note [file ncomms15635-s1.pdf]

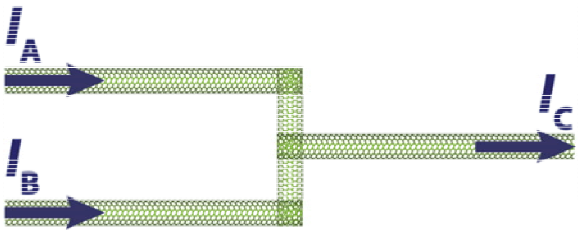

Supplementary Figure 1 | CNT wired-OR gate. The currents through the two input CNTs are joined in the electrically connected junction to perform the logical OR function. The wired-OR output current $I_C = I_A + I_B$, such that I_C propagates a binary 1 if either or both of the input currents represent a 1. Unlike a GNR OR gate, the wired-OR gate consumes the input currents, and can thus be used only once for each logical signal.

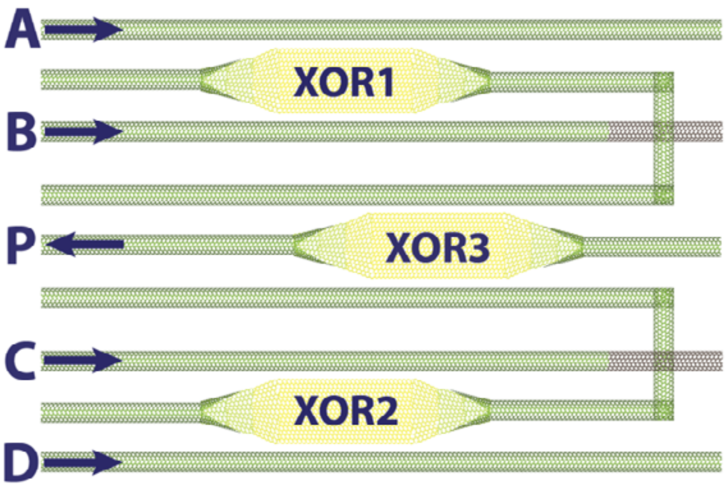

Supplementary Figure 2 | All-carbon spin logic parity gate. The parity gate, used in error detection and correction, computes $P = (A \oplus B) \oplus (C \oplus D) = A \oplus B \oplus C \oplus D$. Thus, when there is an odd number of binary 1 inputs, the output is also 1; the output is 0 if there are an even number of binary 1 inputs.

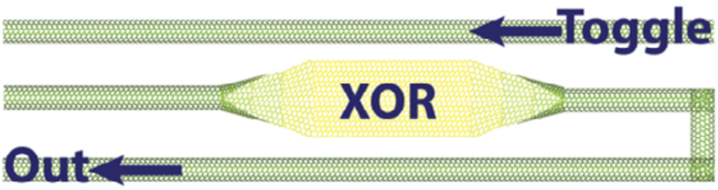

Supplementary Figure 3 | All-carbon spin logic toggle latch circuit. The current through the XOR GNR transistor flows through a control CNT. Therefore, when no current flows through the Toggle input, the control CNT continually reinforces the GNR state. The Toggle input is a pulse of current with significantly long duration to toggle the XOR gate, but shorter than the current propagation time between the XOR and control wire to minimise any unintended interference between the signals.

A	B	C_{IN}	S	C_{OUT}
0	0	0	0	0
0	0	1	1	0
0	1	0	1	0
0	1	1	0	1
1	0	0	1	0
1	0	1	0	1
1	1	0	0	1
1	1	1	1	1

Supplementary Table 1 | Full adder truth table. The one-bit full adder computes the binary addition of two input bits, A and B, and a carry-in bit C_{IN} to compute the sum S and an overflow bit C_{OUT} that is carried to the next digital bit. Several one-bit full adders can be cascaded to perform multi-bit addition, an essential task in modern processors.

Toggle	Out	Out_{NEXT}
0	0	0
0	1	1
1	0	1
1	1	0

Supplementary Table 2 | Toggle latch truth table. The toggle latch operates as a memory circuit, maintaining a state until an input signal is asserted. When the Toggle input pulses a binary 1, the value stored in the Toggle flip-flop is inverted and maintained once the Toggle current is removed.

Supplementary Note 1 | Magnetic instability energy and critical current

To maximise computing efficiency, it is beneficial for the GNR logic gate to switch from the AFM to FM state in response to a current I_C with minimal magnitude. As shown in Fig. 3, this critical switching current is a function of the GNR width, the distance between the GNR and CNT, and the Hubbard U value. In particular, the magnetic instability energy decreases with increased width, and the fields generated by the CNTs are inversely proportional to their distance from the GNR. The simulation results therefore demonstrate that I_C decreases significantly with increasing GNR width and decreasing distance between the GNR and CNT. The Hubbard U parameter, which can be tuned through control of the universal gate voltage, is a further important means of tuning the critical switching current. Though computational resource considerations limited the reciprocal space sampling density and impeded the determination of a precise value for the minimum I_C, it can be readily observed from Fig. 3 that small changes in U induce large changes in I_C.
